# 

**Published:** 1894-06

**Authors:** 


*The following donations have been received since the publication of the list in March:*

*May 31st, 1894.*

F. H. Edgeworth, M.B. (1) ... ... ... ... ... 1 volume.

Arthur B. Prowse, M.D. (2) .. ... .. ... ... 2 volumes.

J. E. Shaw, M.B. (3) ... ... ... ... ... ... .. 1 volume.

E. J. Sheppard (4) ... ... ... ... ... ... ... 2 volumes.

R. Shingleton Smith M.D. (5) ... ... ... ... ... 1 volume.

W. Roger Williams (6) ... ... ... ... ... ... 9 volumes.

### TWELFTH LIST OF BOOKS.

The figures in brackets refer to the figures after the names of the donors, and show by whom the volumes were presented. The books to which no such figures are attached have either been bought from the Library Fund or received through the *Journal.*

**Ball, C. B.** ... ... *The Rectum and Anus* ... ... ... ... ... 2nd Ed. 1894
**Baynton, T.** ... *New Method of Treating Old Ulcers of the Legs.* (4) 2nd Ed. 1799
**Beard and A. D. Rockwell, G. M.** *Medical and Surgical Electricity.* 8th Ed. 1891
**Bigelow, H. R.** ... *An International System of Electro-Therapeutics* ... ... 1894
**Blackwell, Elizabeth** *The Human Element in Sex* ... ... ... ... ... 1894
**Bosworth, F. H.**.. *Diseases of the Nose and Throat.* 2 vols. ... ... 1889-92
**Bouchard, Ch.** ... *Lectures on Auto-Intoxication in Disease.* (Tr. by T. Oliver) ... ... ... ... ... ... ... ... ... ... 1894
**Browning, W. B.**. *Modern Homeopathy*... ... ... ... ... [2nd Ed.] 1894
**Butlin, H. T.** ... *Sarcoma and Carcinoma* ... ... ... ... ... ... ... 1882
,, ... *Malignant Disease of the Larynx* ... ... ... ... ... 1883
,, ... *On the Operative Surgery of Malignant Disease* ... ... 1887
**Carpenter, G.** ... *Congenital Affections of the Heart* ... ... ... ... ... 1894
*Children, An American Text-Book of the Diseases of.* (Ed. by L. Starr and T. S. Westcott) ... ... .. ... ... ... ... 1894
**Cullen, W.** ... ... *Nosology.* [Tr. by C. S.] ... ... ... ... ... (4) 1810
**Dukes, C.** ... ... *On the Features which distinguish Epidemic Roseola from Measles and from Scarlet Fever* ... ... ... ... ... 1894
**Firebaugh, Ellen M.** *The Physician's Wife* ... ... ... ... ... ... ... 1894
**Fresenius, C. R.**.. *Qualitative Chemical Analysis.* (Tr. by A. Vacher). (2) 8th Ed. 1872
,, *Quantitative Chemical Analysis.* (Tr. by A. Vacher). (2) 7th Ed. Vol. I. 1876
**Garrigues, H. J.**.. *Diseases of Women* ... ... ... ... ... ... ... 1894
*German Authors, Clinical Lectures on Medicine and Surgery by.* 3rd Series 1894

**Gull, Sir W. W.** ... *A Collection of Published Writings—Medical Papers.* (Ed. by T. D. Acland) ... 1894
*Gynecology, An American Text-Book of.* (Ed. by J. M. Baldy) ... 1894
**Hall, F. de H.** ... *Diseases of the Nose and Throat* ... 1894
**Heath, C.** ... *Injuries and Diseases of the Jaws.* 4th Ed. (Ed. by H. P. Dean) ... 1894
**Herman, G. E.** ... *Difficult Labour* ... 1894
**Horsley, V.** ... *The Structure and Functions of the Brain and Spinal Cord* ... (1) 1892
**Jeaffreson, C. S.** ... *Nursing in Eye Diseases* ... (5) 1894
**Klein, E.** ... *The Anatomy of the Lymphatic System.* 2 vols. ... 1873–75
*Lexicon of Medicine and the Allied Sciences.* (New Syd. Soc.) P—PIN ... 1893
**Martindale, W.** ... *Analyses of Twelve Thousand Prescriptions* ... 1894
*Medicine, An American Text-Book of the Theory and Practice of.* (Ed. by W. Pepper) ... Vol. II. 1894
**Moure, E. J.** ... *De l'Empyème du Sinus sphénoïdal* ... 1894
,, ... *De l'Extraction des Osselets dans l'Otorrhée* ... 1894
,, **et J. Sabrazès.** *Un Cas d'Angiokératome de la corde vocale droite* 1894
**Parkinson, C. H. W.** *President's Address—Bournemouth Medical Society.* Session 1893-4
**Payne, J. F.** ... *On Seborrhœa and its Consequences* ... 1894
**Praagh, W. Van** .. *Defective Articulation resulting from Cleft Palate.* 2nd Ed. 1894
**Rentoul, R. R.** ... *The Proposed Formation of an Inferior Order of Midwifery Practitioners* ... [1894]
**Rockwell and G. M. Beard, A. D.** *Medical and Surgical Electricity.* 8th Ed. 1891
**Sabrazès et E. J. Moure, J.** *Un Cas d'Angiokératome de la corde vocale droite* 1894
**Saundby, R.** ... *The Common Forms of Dyspepsia in Women* ... 1894
**Sayre, L. E.** ... *Essentials of Practice of Pharmacy* ... 2nd Ed. 1894
**Shaw, J. C.** ... *Essentials of Nervous Diseases and Insanity* ... 2nd Ed. 1894
**Spender, J. K.** ... *Osteo-Arthritis* ... (3) 1889
**Stevens, A. A.** ... *A Manual of Therapeutics* ... 1894
**Treves, F.** ... *The Surgical Treatment of Typhlitis* ... 1890
**Will, J. C. O.** ... *Lectures on Genito-Urinary Diseases* ... 1894

### TRANSACTIONS, REPORTS, JOURNALS, &c.

Alienist and Neurologist, The ... Vol. XIV. 1893
Archives de Neurologie ... Tome XXVI. 1893
Australasian Medical Gazette, The ... Vol. XII. 1893
Bristol Medico-Chirurgical Journal, The ... Vol. XI. 1893
Bristol Port Sanitary District—Annual Report of Medical Officers of Health for 1893 ... 1893
British Journal of Dermatology, The ... Vol. V. 1893
Bulletin de l'Académie de Médecine ... Tomes XXIX. & XXX. 1893
Bulletin de l'Académie Royale de Médecine de Belgique. Tome VII. 1893
Cincinnati Lancet-Clinic, The ... Vol. LXX. 1893
Gazette de Gynécologie ... Tome VIII. 1893
Hospital Annual, Burdett's ... 1894

Hospitals—

Johns Hopkins Hospital Reports, The ... ... Vol. IV., No. 1 1894

Middlesex Hospital—Registrars' Reports for 1880, 1882-89 (6)

Saint Bartholomew's Hospital Reports ... ... ... Vol. XXIX. 1893

Index Medicus ... ... ... ... ... ... ... ... ... ... ... Vol. XV. 1893

International Medical Magazine ... ... ... ... ... ... ... Vol. II. 1894

Johns Hopkins Hospital Bulletin, The ... ... ... Vols. III.-IV. 1892-93

Journal of Nervous and Mental Disease, The ... ... Vol. XVIII. 1893

Journal of Pathology and Bacteriology, The ... ... ... ... Vol. I. 1893

Local Government Board—Further Report and Papers on Epidemic

Influenza, 1889-92 ... ... ... ... ... ... ... ... ... ... ... 1893

Medical and Surgical Reporter, The ... .. ... ... ... Vol. LXIX. 1893

Medical Annual, The ... ... ... ... ... ... ... ... ... ... ... 1894

Medical Chronicle, The ... ... ... ... ... ... ... ... Vol. XIX. 1894

Medical Review ... ... ... ... ... ... ... ... Vol. XXVIII. 1893

Medicinische Bibliographie ... ... ... ... ... ... ... ... ... ... 1893

Medico-Chirurgical Transactions ... ... Vols. LXXIV.—LXXVI. 1891-93

New Zealand Medical Journal, The ... ... ... ... ... ... Vol. VI. 1893

Obstetrical Transactions. Vol. XIII., 1872; Vols. XXVII.—XXXIII., 1886-92

Progrès Médical, Le ... ... ... ... ... ... ... ... ... Tome XVIII. 1893

Registrar-General of Births, Deaths, and Marriages in England,

Annual Reports of... ... ... ... ... ... 1843-45, 1853, 1855-57, 1878

### The William F. Jenks Memorial Prize.

From H. K. LEWIS, London:
*Analyses of Twelve Thousand Prescriptions.* Compiled by W. MARTINDALE. 1894.
*Diseases of the Nose and Throat.* By F. DE HAVILLAND HALL, M.D. 1894.
*The Middlesex Hospital.* Report of the Medical and Surgical Registrars and Pathologist for the year 1892. 1894.

From MACMILLAN & CO., London:
*On the Exact Sensory Defects Produced by a Localised Lesion of the Spinal Cord.* By W. HALE WHITE, M.D. 1893. [Reprint.]

From PARDON & SONS, London:
*Commerce.* No. 31, Vol. II. Jan. 31, 1894.

From YOUNG J. PENTLAND, Edinburgh:
*Handbook of Obstetric Nursing.* By FRANCIS W. N. HAULTAIN, M.D., and JAMES HAIG FERGUSON, M.D. Second Edition. 1894.

From F. J. REBMAN, London:
*Hernia.* By THOMAS H. MANLEY, M.D. 1893.
*Medical Times and Hospital Gazette.* June 2, 1894.

From JOHN MORGAN RICHARDS, London:
*Medical Reprints.* Dec., 1893—May, 1894.

From WILLIAM CHARLES SAMBROOK, London:
*The Cable.* Jan. 20, 1894.

From W. B. SAUNDERS, Philadelphia:
*Diseases of Women.* By HENRY J. GARRIGUES, M.D. 1894.
*A Manual of Therapeutics.* By A. A. STEVENS, M.D. 1894.
*Essentials of Pharmacy.* By LUCIUS E. SAYRE. Second Edition. 1894.
*Essentials of Nervous Diseases and Insanity.* By JOHN C. SHAW, M.D. Second Edition. 1894.

(Per F. J. REBMAN, London):
*A Text-Book of the Theory and Practice of Medicine.* By AMERICAN TEACHERS. Edited by WILLIAM PEPPER, M.D. Vol. II. 1894.
*An American Text-Book of the Diseases of Children.* By AMERICAN TEACHERS. Edited by LOUIS STARR, M.D., assisted by THOMPSON S. WESTCOTT, M.D. 1894.
*An American Text-Book oj Gynecologv.* Edited by J. M. BALDY, M.D. 1894.

From THE SCIENTIFIC PRESS, LIMITED, London:
*A Practical Handbook of Midwifery.* By FRANCIS W. NICOL HAULTAIN, M.D. 1894.
*Lectures on Genito-Urinary Diseases.* By J. C. OGILVIE WILL, M.D. 1894.
*Burdett's Hospital and Charities Annual,* 1894.
*Science Progress.* Vol. I., No. 1. March, 1894.
*The Mother's Help and Guide.* By P. MURRAY BRAIDWOOD, M.D. 1894.

From SIMPKIN, MARSHALL, HAMILTON, KENT & CO., LIMITED, London:
*The After-Treatment of Cases of Abdominal Section.* By CHRISTOPHER MARTIN, M.B. 1894.

From J. W. SIMPSON, Derby:
*Fifth Annual Report of the Derby Borough Asylum,* 1893. 1894.

From SMITH'S PRINTING AND PUBLISHING AGENCY, London:
*Advertising.* Vol. III., No. 5. Feb., 1894.

From SMITH, ELDER & CO., London:
*Spinal Caries.* By NOBLE SMITH. 1894.

From JAMES TILSED, Wimborne:
*Bournemouth Medical Society.* Session 1893-4. President's Address [C H. WATTS PARKINSON.]

From WIEBECK Y TURTL, Buenos Ayres:
*Revista de la Sociedad Médica Argentina.* Vol. II., No. 11. 1893.

From JOHN WRIGHT & CO., Bristol:
*Notes on Nursing in Eye Diseases.* By C. S. JEAFFRESON, M.D. 1894

From 26 RUE HOUDAN, Sceaux:

*Médecine hypodermique.* 6e Année, No 11. Novembre, 1893.

